# TRD-YOLO: A Real-Time, High-Performance Small Traffic Sign Detection Algorithm

**DOI:** 10.3390/s23083871

**Published:** 2023-04-10

**Authors:** Jinqi Chu, Chuang Zhang, Mengmeng Yan, Haichao Zhang, Tao Ge

**Affiliations:** 1 School of Electronic and Information Engineering, Nanjing University of Information Science and Technology, Nanjing 210044, China; 2 Jiangsu Key Laboratory of Meteorological Observation and Information Processing, Nanjing 210044, China

**Keywords:** small object detection, trans, convolution neural network, context aware, LD-Head

## Abstract

Traffic sign detection is an important part of environment-aware technology and has great potential in the field of intelligent transportation. In recent years, deep learning has been widely used in the field of traffic sign detection, achieving excellent performance. Due to the complex traffic environment, recognizing and detecting traffic signs is still a challenging project. In this paper, a model with global feature extraction capabilities and a multi-branch lightweight detection head is proposed to increase the detection accuracy of small traffic signs. First, a global feature extraction module is proposed to enhance the ability of extracting features and capturing the correlation within the features through self-attention mechanism. Second, a new, lightweight parallel decoupled detection head is proposed to suppress redundant features and separate the output of the regression task from the classification task. Finally, we employ a series of data enhancements to enrich the context of the dataset and improve the robustness of the network. We conducted a large number of experiments to verify the effectiveness of the proposed algorithm. The accuracy of the proposed algorithm is 86.3%, the recall is 82.1%, the mAP@0.5 is 86.5% and the mAP@0.5:0.95 is 65.6% in TT100K dataset, while the number of frames transmitted per second is stable at 73, which meets the requirement of real-time detection.

## 1. Introduction

The visual perception technology of intelligent cars is an important part of unmanned driving, with the rapid development of science and technology, traffic sign detection system as a sub-module of intelligent visual perception technology, plays an important role in providing correct traffic signs to improve driving safety, so the recognition of traffic signs has become a hot research topic. However, complex road environments often blur or even distort traffic sign information, and variable light angles can overexpose or darken the image, reducing the visibility of traffic signs. These situations seriously affect the accuracy and speed of traffic sign detection. Therefore, it is necessary to design a new detection network to maintain high accuracy and high detection speed in complex environments.

Most of the existing traffic sign recognition (TSR) [1] algorithms are divided into two types: traditional algorithms and deep learning algorithms. Traditional algorithms often detect traffic signs through shape features, edge features, and color features. For instance, utilizing HSV and HSI [2] to match color features in the identification of traffic signs or employ histograms of oriented gradients (HOG) [3] and scale-invariant feature transform (SIFT) [4] to detect shape features of traffic signs; these algorithms can detect traffic signs in simple environments, but because their ability to extract features is weak, they are unable to fulfill the demands of small traffic sign detection tasks in complicated background. While the object detection algorithm based on deep learning has gradually become the mainstream method in traffic sign detection due to its powerful feature extraction capability.

**Figure 1:**
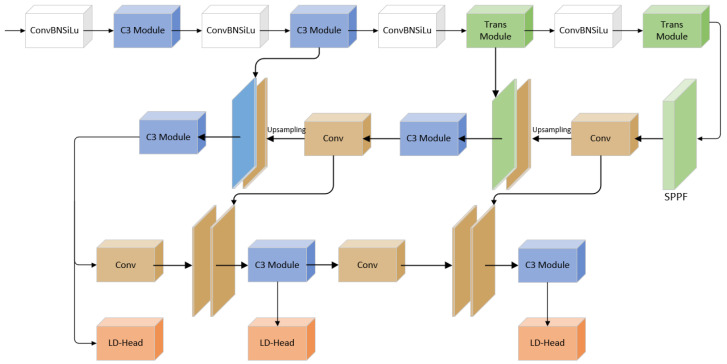
The architecture of TRD-YOLO.

Existing deep learning-based object detection algorithms can be divided into two categories: one-stage detection algorithms and two-stage detection algorithms. R-CNN [5], SPPNet [6], Fast RCNN [7], etc., are examples of typical two-stage detection algorithms; SSD [8], RetinaNet [9], YOLO [10], etc., are examples of typical one-stage detection techniques. Zuo et al. [11] applied Faster R-CNN to traffic sign detection. The YOLOv3 network was introduced by Shehan P. Rajendran et al. [12] for the purpose of detecting traffic signs. To better detect traffic signs, Li et al. [13] integrated an attention mechanism with a YOLOv4 network. These algorithms have achieved some achievements in the task of traffic sign detection, but due to the small size of traffic signs, variable imaging angles, and complex lighting environment in real scenarios, the detection accuracy and speed still cannot reach a reasonable level. YOLOv5 [14] is a network with superior overall performance, but the accuracy of detecting small traffic signs in complex environments is low. To address these problems, this paper proposes a new network based on YOLOv5s, called TRD-YOLO (Trans-Decoupled YOLO), the structure of which is shown in Figure 1. Fusing the Transformer mechanism [15] and convolutional neural network (CNN) and proposed a new lightweight decoupled head to enhance the output capability of the network.

Compared with YOLOv5, the proposed model has a good detection effect on traffic signs in complex environments, such as deformation, occlusion, and dimness. It can greatly improve the problems of inefficient traffic sign recognition, missed detection, and false detection.

Our main contributions are as follows:

1. In order to solve the problem of complex background inhibition backbone network extracting feature information, this paper introduces the Transformer mechanism into the backbone network, replaces part of the convolution layer with the designed Trans module, enables the network to better detect small objects by focusing on global contextual information, and uses self-attention to learn salient feature information more efficiently.
2. Since the detection head of YOLOv5 combines the classification task with the regression task for parsing output, which affects the performance of the network for the detection of small traffic signs in complex environments, we refer to the design idea of Decoupled Head in YOLOX [16], and consider the problem of network complexity and real-time detection, designing a new detection head called Lightweight Decoupled Head (LD-Head), which suppresses the interference of redundant features, separates the classification task from the regression task, and better parses the feature information extracted by the network for output.
3. We propose a new model, TRD-YOLO, for small traffic sign detection tasks in complex environments.

On the TT100K dataset, our model TRD-YOLO improves accuracy by 2.6%, recall by 6.7%, mAP_0.5 by 6.4%, and mAP_0.5:0.95 by 6.8% compared to YOLOv5.

## 2. Related Work

### 2.1. YOLOv5s

In recent years, CNN-based object detection algorithms have become the mainstream due to the rise of deep learning. YOLO, as one of the most classic object detection models, has been proposed by Redmon et al. [10] since 2016 and has been a hotspot for researchers. Zhang et al. [17] proposed an improved model based on YOLOv2 for detecting traffic signs. Wang et al. [18] combined the small object detection layer with YOLOv4 network for traffic sign detection. Yan et al. [19] combined attention with YOLOv5 and proposed a new model for traffic sign detection. These methods have inspired us tremendously. However, the YOLO series of algorithms are mainly used to detect general objects, and the detection ability of small objects is weak. Therefore, in the face of traffic sign detection in complex road scenarios, this paper proposes TRD-YOLO, to improve the detection accuracy of small traffic signs by using YOLOv5s algorithm as the basic framework.

### 2.2. Small Object Detection

There are usually two definitions of small objects, one is relative size definition, that is, the target size is less than 10% of the original image size, the other is the definition of absolute size, that is, the target size is less than 32 × 32 pixels. Therefore, in object detection tasks, small object detection is usually a challenging task. At present, the improvements made for small object detection are divided into the following types: multi-scale detection, high-resolution, context-aware.

For context-aware, there are several methods, just like the FPN [20] and PAN [21], which use the top–down, bottom–up paths to fuse the features of different layers. In this paper, we use the FPN+PAN structure as the feature fusion module of the network. The Transformer mechanism has also been used in the backbone of the model to enhance the perception of context, which is a new attempt. In the meantime, a more powerful prediction head can also affect the detection results of the small object.

## 3. The Architecture of TRD-YOLO

In this study, an effective traffic sign detection algorithm TRD-YOLO is proposed. The algorithm improves on two parts of YOLOv5: backbone network and prediction head. This section first describes how the Trans module added to the backbone works, then describes the design principles of Lightweight Decoupled Head, and explains the advantages of both modules.

### 3.1. Context-Aware Module

**Figure 2:**
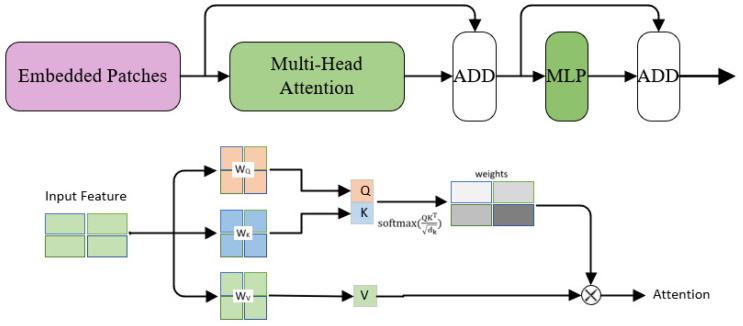
Trans module, above is the overall process, below is the Attention calculation process.

YOLOv5 does not have an overall perception of the contextual information of the feature map, which results in some features being discarded. After investigation [22,23], the Transformer is a model proposed by Google in 2017 for application in the field of NLP, which has an excellent performance in several tasks. Inspired by this, we designed the Trans Module to encode 2D images, whose structure is shown in Figure 2. The ability of the Trans module to perceive global feature information can compensate for the shortcomings of insufficient feature extraction in CNN, and the Trans module can use the self-attention mechanism to focus on more representative features, which greatly increases the context-aware capabilities of the model. Combined with the advantages of CNN in extracting visual features at the bottom, the new backbone can have better performance in object detection tasks.

At the input, we reshape the 2D image information ($x\in R^{\left(H\times W\times C\right)}$) into a sequence ($x_{P}\in R^{\left[D\times \left(p^{2}\cdot C\right)\right]}$), (H,W) represent the resolution of the input image, C is the number of channels, and D is the valid input sequence length of the Transformer encoded portion. These sequences are sent to the Transformer’s encoder for encoding. As shown in Figure 2, Q, K, and V represent query, key, and value in transformer theory, W is a matrix that can be learned, $d_{k}$ represents the dimension of K. K and Q are calculated by Equation (1) to obtain weight coefficients.

(1) $$softmax(\frac{QK^{T}}{\sqrt{d_{k}}})$$

Then, make a dot product between weights and V to obtain the enhanced output feature (Attention), which is the key expression of contextual information. Meanwhile, to make attention-manipulated location awareness, using standard learnable 1-D position embeddings (turn a 2D image into a 1D sequence) with linear layers to preserve position information, and finally into MLP (two fully connected layers) to classify the image. In the meantime, the input and output are connected by residual connections to avoid the degradation problem of the deep network.

Among the structure, multi-head attention is the core layer of the module, which is equivalent to being integrated by multiple different self-attention, so the multi-head attention can be balanced according to different weights, so that the network can learn more diverse feature information.

Based on experimental analysis, we add the Trans module to the deep backbone network to form a new backbone TRCSP. Because in the shallow network layer, the resolution of the feature map is too large, the computational cost and memory cost caused by global feature extraction are very large. More importantly, the semantic information in the shallow features is not rich, so the Trans module cannot play a good role in enhancing the correlation between the semantic information, but may interfere with and lose some enriched semantic information instead. While in the deep network, the feature map resolution is low and contains rich semantic information, which can better utilize the advantages of the Trans module in extracting global features while saving a lot of memory resources and computing resources.

### 3.2. Lightweight Decoupled Head

According to the literature [24,25], in the regression and classification tasks of object detection, there exists a problem of spatial dislocation, that is, the classification task is more concerned with which category the extracted features are closer to, while the regression task pays more attention to the distance between the prediction box and the Ground-Truth box to correct the location of the bounding box. In this paper, the benchmark model YOLOv5 uses the coupled detection head, which combines the classification and localization tasks into one output. It lacks task-specific learning capability, which damages the detection capability of network. In response to these problems, Decoupled Head is used in YOLOX, which separates different tasks into different paths for output, obtaining certain performance improvements.

**Figure 3:**
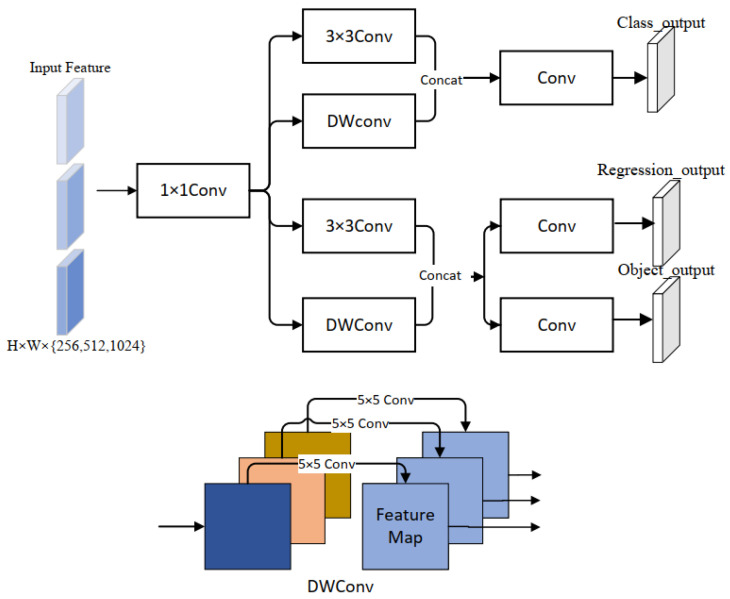
Lightweight decoupled head, H represents height, W represents width.

However, according to our experimental analysis, the multiple multi-level convolutional tandem structures of Decoupled Head in YOLOX increase the complexity of the network structure, and, in general, deep neural networks extract a large amount of redundant feature information during the feature extraction process, especially in deep networks, wasting a lot of memory resources and computing resources, which interferes with the process of learning features. To address this problem, this paper designs a Lightweight Decoupled Head (LD-Head) to parse the output, suppress the effects of redundant features, and improve the accuracy of detection. In the face of small traffic signs in complex environments, it has better detection performance and stronger robustness. The structure of the Lightweight Decoupled Head is shown in Figure 3.

We use Depthwise convolution (DWConv) [26] to suppress redundant features while lightweight the network structure. Its calculation with normal convolution is compared as follows. Suppose the size of the input feature map is $D_{H}\times D_{W}\times M$, the size of the convolution kernel is $D_{F}\times D_{F}\times M$, and the quantity is N. So the total computation of N convolutions is shown in Equation (2):

(2) $$D_{H}\times D_{W}\times D_{F}\times D_{F}\times M\times N$$

Depthwise convolution is composed of channel-by-channel convolution. Depthwise convolution needs to convolve each channel of the input, and only one convolution kernel is used per channel, so the convolution kernel size of deep convolution is $D_{F}\times D_{F}\times 1$, the number of output channels is consistent with the number of input channels, is M, and the calculation amount is shown in Equation (3):

(3) $$D_{H}\times D_{W}\times D_{F}\times D_{F}\times M$$

It can be seen from depthwise convolution that the features of different channels are separated from each other, this greatly reduces the amount of computation.

The gap between the two calculated quantities can be clearly seen from Equations (2) and (3), and the use of deepthwise convolution can significantly reduce the computation amount. The compression of the model is shown in Equation (4):

(4) $$\frac{1}{N}$$

The LD-Head separates the classification task from the regression task and has three output detection heads in total: class_output, regression_output, and object_output. At the input, we reduce the dimension of the channels by using a 1 × 1 convolutional layer and unify the number of output channels, then separate the tasks through two parallel subnets. Each subnet has two input branches, and the number of input channels per branch is 1/2 of the number of output channels of the previous layer. In a branch, we use 3 × 3 convolution to preserve rich feature information, and improve the expression ability of the network. In the other branch, using the DWConv to extract features, suppress the impact of redundant features and reduce network complexity. We will fuse the feature maps of the two branches for output. Among the two subnets, class_output belongs to the classification subnet, and this branch mainly predicts the class of objects in the bounding box; Regression_output and object_output belong to the regression subnet, where regression_output mainly detects the coordinate information (x, y, w, h) of the target box, and object_output mainly determines whether the bounding box is foreground or background. Finally, we stitch together the classification and regression results by channel dimension to obtain the output feature.

### 3.3. Data Preprocessing

For the task of detecting small traffic signs in complex environments, we propose some data augmentation methods to enrich the dataset, such as Mosaic [27] and Mixup [28]. Considering that most of the images are in sufficient lighting and lack the experimental environment in a dim environment, so we expanded some of the dataset pictures by adjusting the brightness and saturation of the pictures.

## 4. Experimental Analysis

### 4.1. Datasets

**Figure 4:**
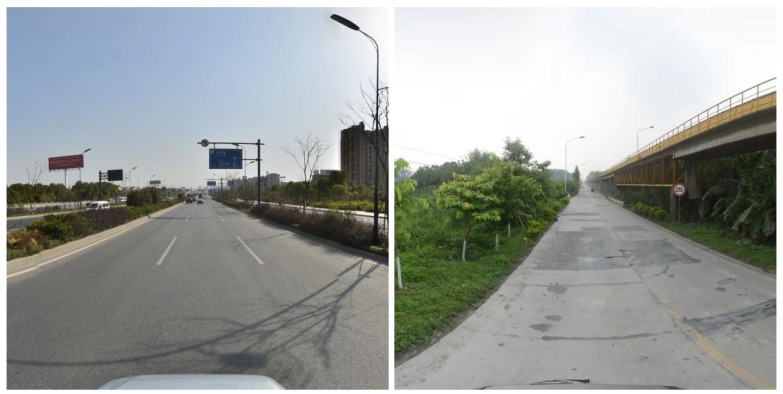
Images in TT100K.

TT100K dataset is a large-scale traffic sign dataset jointly produced by Tsinghua University and Tencent [1]. Compared with GTSDB [29] (German traffic sign dataset) and CCTSDB [17] (Chinese traffic sign detection dataset), the dataset contains a large number of road traffic signs in various complex environments and weather conditions, very small in size, and very close to practical application scenarios, which is extremely difficult to detect. Some examples are shown in Figure 4.

**Figure 5:**
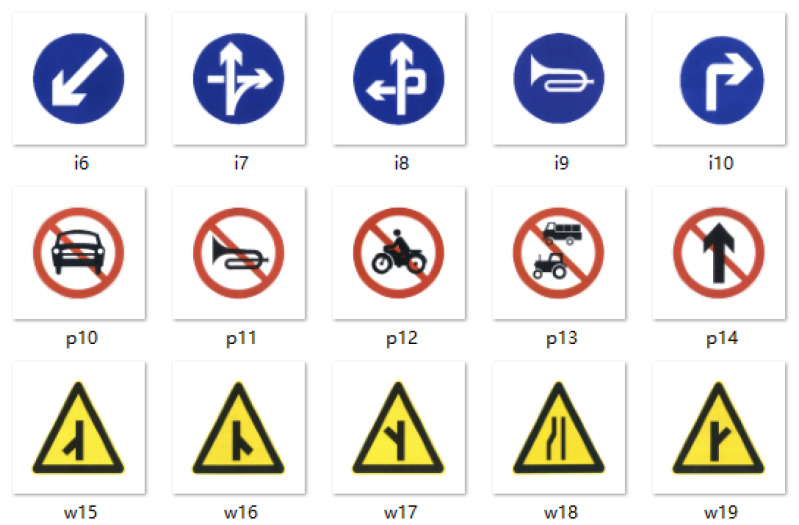
TT100K dataset.

TT100K dataset contains 221 categories of traffic signs, and the resolution of each data sample is 2048 × 2048. The original dataset has a total of 6107 training data and 3073 test data. According to the experimental analysis, there are a large number of traffic signs with a very low proportion in the native dataset, which cannot be learned effectively. To address this problem, we analyse the number of various types of traffic signs in the dataset, and select 45 categories of traffic signs with a number more than 100 to balance the sample discrepancy caused by different categories of traffic signs in the dataset. The example of the dataset is shown in Figure 5, and the category names are shown in Table 1, where the signs starting with “w” are warning signs, the signs starting with “p” are forbidden signs, the signs starting with “i” are indicating signs, and the flags ending with * indicate the numerical value, such as pl50, pl60, and pl100.

In the end, a total of 7965 images were selected, of which the training set contains 5291 images and the test set contains 2674 images.

### 4.2. Evaluation Criteria

The hardware platform for this experiment is an Intel(R) Core(TM) i7-12400KF CPU, 32 GB of RAM, and NVIDIA GeForce GTX3060 12 G graphics card, and the operating system is Windows 10.

In order to objectively evaluate the detection performance of the algorithm in complex road scenarios, this paper uses a variety of evaluation criteria to verify the proposed model from different perspectives. Precision represents the probability that the sample that is predicted to be a positive sample is correctly predicted in the prediction result. Recall represents the probability that in the positive sample of the original sample, it will finally be correctly predicted as a positive sample. The precision and recall are shown in Equations (5) and (6), TP represents the number of correctly detected samples, FP represents the number of falsely detected samples, and FN represents the number of samples that missed detection. AP is the accuracy of a single class, and mAP is the average AP of all classes, defined in Equations (7) and (8).

(5) $$Precision=\frac{TP}{TP+FP}$$

(6) $$Recall=\frac{TP}{TP+FN}$$

(7) $$AP=\int_{0}^{1}P\left(r\right)dr$$

(8) $$mAP=\frac{1}{N}\sum_{1}^{N}AP_{i}$$

### 4.3. Experimental Details

In this article experiment, we set the input image size of 640 × 640, the batch size set to 8, num_workers set to 6, use the SGD as optimizer, with a weight decay of 0.0005 and momentum of 0.937 as default. At the beginning of training, we first perform warm-up training for three epochs, where the warmup_momentum is set to 0.8, and use one-dimensional linear interpolation to update the learning rate of each iteration. After warm-up training, the cosine annealing function is used to attenuate the learning rate, where the initial learning rate is 0.02 and the minimum learning rate is 0.2 × 0.01. Finally, we trained the model for 600 epochs.

## 5. Experimental Results and Analysis

Table 2 shows our results evaluated on the TT100K dataset. We selected several classic models to conduct comparison experiments with our proposed TRD-YOLO, including many excellent YOLO family algorithms and its improved algorithms. As can be seen from Table 2, TRD-YOLO’s superior performance with mAP@0.5 is 86.5%, even 6.4% above the benchmark YOLOv5s, proves the effectiveness of our improvements.

Table 3 shows the experimental detection results, as well as the evaluation metrics for precision, recall, and AP for each class in the dataset. Among them, forbidden signs improved the most, followed by warning signs. In addition, for the AP values of each category of the dataset, the AP values of the proposed algorithm for indicating traffic sign category, forbidden traffic sign class, and warning traffic sign class are 94.4%, 79.9%, and 85.2%, respectively, which are improved by 3.8%, 8.5%, and 6.9% compared with the baseline.

**Figure 6:**
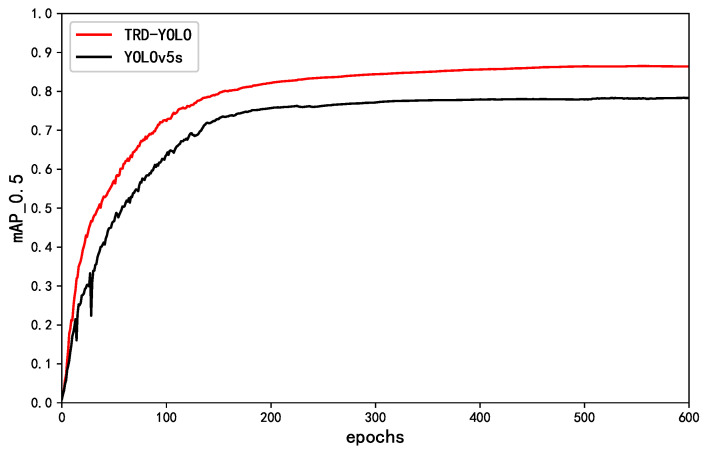
Comparison chart of mAP of YOLOv5s and our method.

Figure 6 shows the comparison of the training process between the TRD-YOLO model and the YOLOv5s model, from which it can be seen that TRD-YOLO has strong learning ability at the beginning of training, and the training process is faster and smoother than convergence. The above comparison shows that the proposed method has good performance.

### 5.1. Ablation Experiment

To further verify the validity of our proposed model, we performed ablation experiments on the TT100K dataset. Due to the superior comprehensive performance of the classic model YOLOv5s, we use YOLOv5s as our baseline to verify the improvement effect of each module proposed by us. The results are shown in Table 4. “Kmeans” is a clustering algorithm [32], “TRCSP” represents our proposed new backbone extraction network for CSPDarknet fusion Trans module. “D-Head” represents the decoupled head that we separated from YOLOX. “LD-Head” represents the new lightweight decoupled head that we proposed.

**Figure 7:**
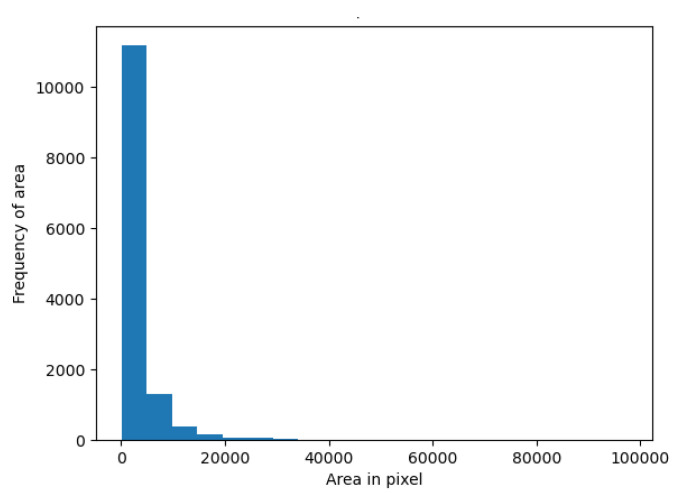
Statistical chart of the data sample area.

As can be seen from Table 4, each innovation point has improved on the baseline. In this paper, the data sample distribution is shown in Figure 7, it can be seen that the resolution of most traffic signs is concentrated at 100 × 100, compared to the input size of 2048 × 2048, the size is extremely small, so we need the kmeans algorithm to recalculate the size of the prior box according to the dataset.

Our new priori bounding box has a significant improvement in all evaluation indicators, with mAP@0.5 improving by 3.3%, proving that the new priori bounding box is more suitable for small traffic signs. TRCSP solves the problem of limited receptive field of convolutional neural network. It can better detect objects by combining the global feature information of the image, with 4.9% improvement in mAP@0.5. The D-Head is a comparative experiment we perform, focusing on a comprehensive comparison with the LD-Head we proposed. Compared with baseline, the LD-Head proposed by us has improved in all indicators, with precision improved by 2.6%, recall increased by 6.7%, mAP@0.5 increased by 6.4%, mAP@0.5:0.95 improved by 6.8%. At the same time, compared with the structure using D-Head in the same environment, the accuracy is improved by 1.1%, the mAP@0.5 is improved by 0.4%, and the mAP@0.5:0.95 is improved by 0.4%, which verifies that LD-Head has better performance in detecting small traffic signs in complex environments.

**Figure 8:**
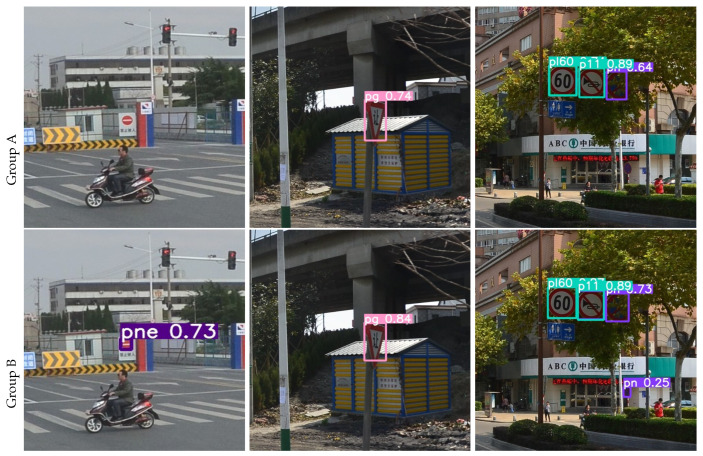
Detection in daytime scenes. The **left** column shows the missed detection; the **middle** column is deformed traffic sign detection; and the **right** column is occlusion traffic sign detection.

**Figure 9:**
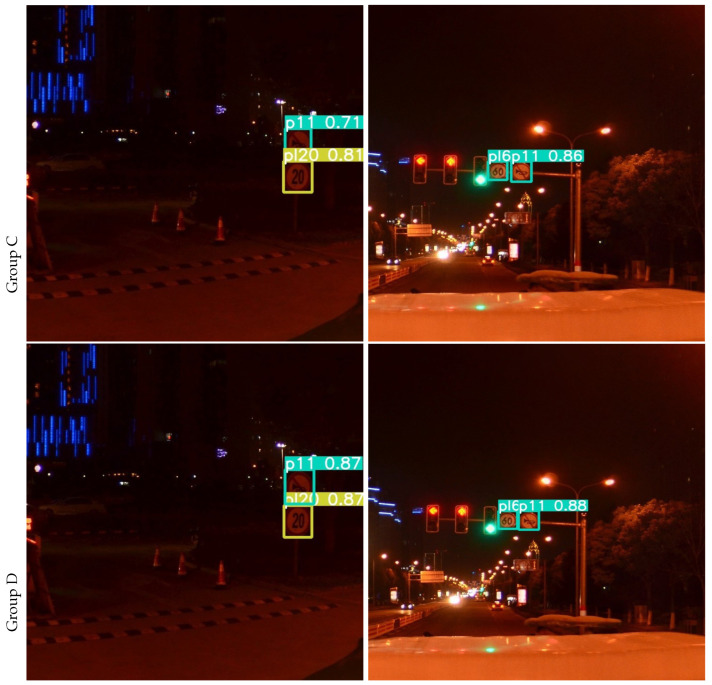
Detection in night scenes.

The results of the ablation experiment on the TT100K dataset are visualized as shown in Figure 8 and Figure 9. Group A and C pictures show the detection results of YOLOv5s, the picture in Group B and D shows the TRD-YOLO detection results. When traffic signs are very small, deformed, occluded, or in night scenes, YOLOv5s often have problems with missed detection, false detection, or low confidence, while TRD-YOLO can accurately locate and classify each traffic sign and detect it with high confidence. As shown in the left column, YOLOv5s missed a “pne” traffic sign, while TRD-YOLO accurately detected it. As shown in the middle column, when faced with deformed traffic sign, YOLOv5s cannot judge it with certainty, while TRD-YOLO detects it with a confidence rate of 0.84, which is 0.1 higher than YOLOv5s. As shown in the right column, when the traffic sign is backlit or occluded, the detection capability of YOLOv5s is greatly reduced, will missed detection and with low confidence, while TRD-YOLO can extract richer features with the ability of global feature extraction, enhance context aware and rely on a more powerful decoupled head for detection. As can be seen from Figure 9, the detection ability of YOLOv5s is weaker than that of TRD-YOLO in the night scene.

### 5.2. Detection Speed Experiments

In order to ensure the real-time detection of traffic signs, we comprehensively investigate the real-time detection performance of the model from four directions: number of parameters, calculations, detection speed, and mAP@0.5. As shown in the Table 5, the parameter growth and computational power requirements from D-Head are enormous. In response to this problem, our novel LD-Head solves this problem perfectly. At the cost of small-increased parameters and calculations, the FPS requirements for real-time detection are guaranteed, and better detection results are also achieved.

To further verify the validity of TRD-YOLO, we enumerate the changes of some evaluation indexes of traffic sign detection, as shown in Table 6, to more clearly demonstrate the results of the experiments.

## 6. Conclusions

This paper focuses on improving the detection performance of small traffic signs in complex environments. Although there have been good detection methods before, the complexity and accuracy of the model still needs to reach a reasonable level. For this, a high-performance object detection model, TRD-YOLO, is proposed for the detection of small traffic signs in complex environments. In the backbone feature extraction phase, we fused the Transformer mechanism with CNNs, the ability of global feature extraction is introduced, enhancing the correlation between information and the respective advantages of the two mechanisms are fully integrated. In the detection head, we propose a parallel lightweight decoupled head that separates the classification task from the regression task for resolution, the parallel design enriches the feature information as much as possible, and the lightweight design suppresses redundant feature interference while speeding up the detection speed. We verified the performance of TRD-YOLO through transverse comparison experiments, ablation comparison experiments, and detection speed experiments, the TRD-YOLO is able to detect small traffic signs with high accuracy in complex road scenarios, and all evaluation criteria are improved, with the final improvement of 2.6% in Precision, 6.7% in Recall, 6.4% in mAP@0.5, and 6.8% in mAP@0.5:0.95. In future work, we plan to design high-performance traffic sign detection algorithms for edge devices. In addition, we intend to deal with special weather traffic sign detection, such as rain, snow, and fog, in future work.

## Figures and Tables

**Table 1:** The 45 types of traffic signs used in the experiment.

Category	Sign Name
Indicating signs	i2,i4,i5,il100,il60,il80,io,ip
Forbidden signs	p10,p11,p12,p19,p23,p26,p27,p3,p5, p6,pg,ph4,ph4.5,ph5,pl100,pl120,pl20, pl30,pl40,pl5,pl50,pl60,pl70,pl80,pm20, pm30,pm55,pn,pne,po,pr40
Warning signs	w13,w32,w55,w57,w59,wo

**Table 2:** The detection performance comparison of different methods on the TT100K dataset.

Models	Input Size	Backbone	FPS	Map@0.5 (%)
SSD [8]	300 × 300	VGG16	53	59.8
YOLOv3 [30]	416 × 416	Darknet	50	74.3
YOLOv4-Tiny [31]	640 × 640	CSPDarknet	131	76.8
YOLOv5n [14]	640 × 640	CSPDarknet	169	76.9
YOLOv5s	640 × 640	CSPDarknet	136	80.1
Yan et al. [19]	640 × 640	CSPDarknet	87	83.5
YOLOX [16]	640 × 640	CSPDarknet	55	84.9
TRD-YOLO	640 × 640	TR-CSPDarknet	73	86.5

**Table 3:** Comparison of different types of traffic signs.

Models	Class	Precision (%)	Recall (%)	AP (%)
	Indicating signs	90.6	87.1	90.6
YOLOv5s	Forbidden signs	74.4	66.8	71.4
	Warning signs	86.1	72.3	78.3
	Indicating signs	92.8	91.7	94.4
TRD-YOLO	Forbidden signs	79.5	77.4	79.9
	Warning signs	86.6	77.2	85.2

**Table 4:** Ablation experiment based on baseline.

Methods	Kmeans	TRCSP	D-Head	LD-Head	Precision (%)	Recall (%)	mAP@0.5 (%)	mAP@0.5:0.95 (%)
YOLOv5s					83.7	75.4	80.1	58.8
YOLOv5s	✓				83.8	80.5	83.4	63.2
YOLOv5s	✓	✓			85	80.2	85	64.3
YOLOv5s	✓	✓	✓		85.2	82	86.1	65.2
YOLOv5s	✓	✓		✓	86.3	82.1	86.5	65.6

**Table 5:** Real-time detection evaluation.

Models	Params(M)	GFLOPS	FPS	mAP@0.5 (%)
YOLOv5s	7.1	16.4	136	80.1
YOLOv5s + TRCSP	7.1	15.4	87	85
YOLOv5s + TRCSP + D-Head	14.4	56	52	86.1
YOLOv5s + TRCSP + LD-Head	12.6	26	73	86.5

**Table 6:** Some categories of evaluation indicators, bold values indicate the current optimal values.

Category	Precision (%)	Recall (%)	mAP@0.5 (%)	mAP@0.5:0.95 (%)
i2				
——YOLOv5s	0.9	0.786	0.841	0.601
——TRD-YOLO	**0.9**	**0.891**	**0.926**	**0.685**
il80				
——YOLOv5s	0.946	0.883	0.967	0.78
——TRD-YOLO	**0.986**	**0.932**	**0.974**	**0.793**
p5				
——YOLOv5s	0.878	0.785	0.898	0.723
——TRD-YOLO	**0.844**	**0.911**	**0.925**	**0.741**
p6				
——YOLOv5s	0.865	0.448	0.632	0.495
——TRD-YOLO	**0.852**	**0.69**	**0.869**	**0.648**
pg				
——YOLOv5s	0.888	0.928	0.905	0.651
——TRD-YOLO	**0.866**	**0.938**	**0.931**	**0.721**
w13				
——YOLOv5s	0.91	0.821	0.839	0.511
——TRD-YOLO	**0.912**	**0.893**	**0.926**	**0.656**
w59				
——YOLOv5s	0.806	0.858	0.887	0.649
——TRD-YOLO	**0.859**	**0.968**	**0.966**	**0.722**

## Data Availability

Not applicable.
